# Association of the Serum Vascular Endothelial Growth Factor Levels With Benign Prostate Hyperplasia and Prostate Malignancies

**DOI:** 10.5812/numonthly.14778

**Published:** 2014-04-27

**Authors:** Mohammad Reza Sharif, Amirreza Shaabani, Hossein Mahmoudi, Hassan Nikoueinejad, Hossein Akbari, Behzad Einollahi

**Affiliations:** 1 Department of Pediatrics, Kashan University of Medical Sciences, Kashan, IR Iran; 2 Department of Urology, Kashan University of Medical Sciences, Kashan, IR Iran; 3 Department of Nephrology and Urology Research Center, Baqiyatallah University of Medical Sciences, Tehran, IR Iran; 4 Department of Biostatistics, Kashan University of Medical Sciences, Kashan, IR Iran

**Keywords:** Vascular Endothelial Growth Factor A, Prostatic Neoplasms, BPH

## Abstract

**Background:**

Recently, the development of new biomarkers as prognostic and predictive markers in prostate cancer has been crucial.

**Objectives:**

This study was aimed to determine whether serum vascular endothelial growth factor (VEGF) levels would be a prognostic marker or risk assessment factor in patients with prostate cancer and to investigate whether it could differentiate cancerous tissue from benign prostate hyperplasia (BPH).

**Patients and Methods:**

We enrolled 44 patients with prostate cancer, 57 patients with BPH, and 57 healthy individuals. Serum VEGF levels was measured by ELISA and was compared among all groups; then, its correlation with PSA and Gleason score in cancerous group was assessed. In addition, by using receiver operating characteristic (ROC) curve and area under curve (AUC), we determined the sensitivity and specificity of VEGF as well as combined variable of VEGF and PSA as a diagnostic marker of prostate cancer.

**Results:**

Serum VEGF level was significantly higher in patients with prostate cancer in comparison to the other groups (P value < 0.001); however, it was not different between BPH and control groups. Only in cancerous group a significant correlation between VEGF and PSA was found (r = 0.425, P = 0.004). Assessing the risk of prostate cancer, we found a powerful correlation between the VEGF alone as well as the combination of VEGF and PSA with prostate cancer.

**Conclusions:**

VEGF may be a diagnostic biomarker of prostate cancer. In addition, it may differentiate the cancerous tissue from BPH. We suggest that VEGF combined with PSA may be used as a screening test of prostate cancer.

## 1. Background

Prostate cancer is the fourth cause of death in men worldwide (9.2%) being surpassed only by lung cancer (18%). Due to the magnitude of the impact over public health, strong efforts have been made aiming the prevention, early diagnosis, and treatment of this disease (1).

As we enter into the postgenomics era, novel biomarkers of prostate cancer will invariably emerge (2). Angiogenesis is a critical component of prostate cancer development and progression (2, 3) and some studies have shown a close association between increased microvascular density and Gleason score (an evaluating system for prostate cancer based on microscopic features of prostate cells) in advanced prostate cancer (2).

Vascular endothelial growth factor (VEGF), which is expressed at elevated levels in several tumor types, is an important hypoxia-inducible proangiogenic protein and a potent inducer of endothelial cell growth (4, 5). The clinical importance of VEGF in the concept of tumor growth is supported by the fact that most tumors produce VEGF and inhibition of VEGF-induced angiogenesis significantly reduces tumor growth in vivo (6). Although the serum levels of VEGF in many types of cancer have been correlated with stage of the disease (7), the validity of the VEGF as a prognostic marker of prostate cancer is controversial. Some studies stated that the plasma (8) as well as urinary (9) levels of this factor had a prognostic value in both localized (8, 10, 11) and metastatic (8, 9) prostate cancer, especially in early stages of the tumor (12, 13). In addition, few studies have introduced the value of VEGF as a screening test for prostate cancer, as a tool for its staging, as a target for therapeutic strategies (14), and as the only significant prognostic factor of disease-specific survival (5).

On the other hand, some studies revealed that the expression of VEGF was not correlated with any clinicopathological parameters among prostate cancer specimens (15). It was stated that the plasma levels of VEGF did not have any association with the clinical staging (3, 7), the form (benign vs. malignant) (16) and the progression (localized vs. metastatic) (17) of the prostate cancer. These controversies are also seen in benign prostatic hyperplasia (BPH). Some researchers came to the conclusion that serum level of VEGF as well as its expression is increased in the benign patterns like BPH (4) but others believed that its expression would decrease along with progression of the disease (15).

## 2. Objectives

For the first time in Iranian population, we aimed to compare the serum levels of VEGF in patients with prostate cancer with the levels in patients with BPH as well as healthy people and to determine whether it would be a prognostic marker as well as differentiating factor in such patients.

## 3. Patients and Methods

This study was performed under KAUMS (Kashan University of Medical Sciences) Ethics Committee-approved protocol and written informed consent was obtained from all participants. The study protocol conformed to the ethical guidelines of the 1975 Helsinki Declaration. Exclusion criteria consisted of prior surgery or any other treatment of prostate cancer or BPH as well as any active infection or inflammatory process. From May 2011, serum samples were collected from 158 men. Forty-four of them were new cases of prostate cancer with no history of surgical or medical procedures at the time of sampling, 57 men were new cases of BPH based on clinical and sonographic criteria (diffused enlargement of transitional zone of prostate nearby the base of bladder with nodular and heterogenic echo). We recruited 57 age-assimilated healthy control men without any complaint or sign of inflammatory/noninflammatory diseases. In those with an abnormal rectal exam and a prostate-specific antigen (PSA) > 4, we proved the diagnosis of cancer by biopsy. We considered just new cases without any surgical procedures to assess TMN; therefore, it was impossible to evaluate the staging of the patients. Five patients with BPH attended with a mildly elevated PSA levels; therefore, we confirmed no evidence of malignancy in them with histological findings on the biopsy specimens.

All patients and controls had a blood sample drawn into a tube with no anticoagulant. In BPH, patients’ blood samples were taken after the diagnosis and before any treatment, and in cancer suspects (according to rectal exam findings and PSA) before prostatic biopsy. Blood samples of biopsy-confirmed cancerous patients were enrolled in the study. Therefore, nobody had any surgical and medical treatment at the time of sampling. The serum stored at −20°C until analysis. Concentration of serum VEGF was measured using a quantitative immunoassay technique, namely enzyme-linked immunosorbent assay (ELISA), by a commercially available ELISA kit (eBioscience, USA), according to the manufacturer’s instruction. Samples were analyzed twice and mean VEGF levels reported in picogram per milliliter in each group. All data were analyzed by SPSS v.17 (SPSS Inc, Chicago, Illinois, USA) using descriptive statistics, namely chi-square and fissure exact tests. Analytical data were analyzed via ANOVA and student t-test. We used a logistic regression model to assess the effective factors on prostate cancer risk. Then the impact of all factors on VEGF was studied simultaneously by covariance analytic model. Using receiver operating characteristic (ROC) curve and area under curve (AUC), we tried to determine the sensitivity and specificity of VEGF as a diagnostic marker of prostate cancer. Moreover, using analysis of the principle components, we calculated linear combination of VEGF and PSA variables with equal co-efficiencies. Finally, we calculated the specificity, sensitivity and AUC of combined variable to predict prostate cancer.

## 4. Results

Table 1 shows the mean ages, serum levels of VEGF as well as PSA, and prostate volume in each group. The serum level of VEGF and PSA were significantly higher in the cancer group in comparison to the BPH and healthy groups (P < 0.001).

Table 2 shows the correlation coefficient of VEGF with Gleason score, PSA, and prostate volume. There was a meaningful correlation between VEGF and PSA in prostate cancer group (r = 0.425, P = 0.004). Using a logistic regression model, we determined that the age (P < 0.001) and VEGF (P < 0.001) as risk factors of prostate cancer. Adjusting the age effect, VEGF had an association with prostate cancer risk (P < 0.001).

**Figure 1.**
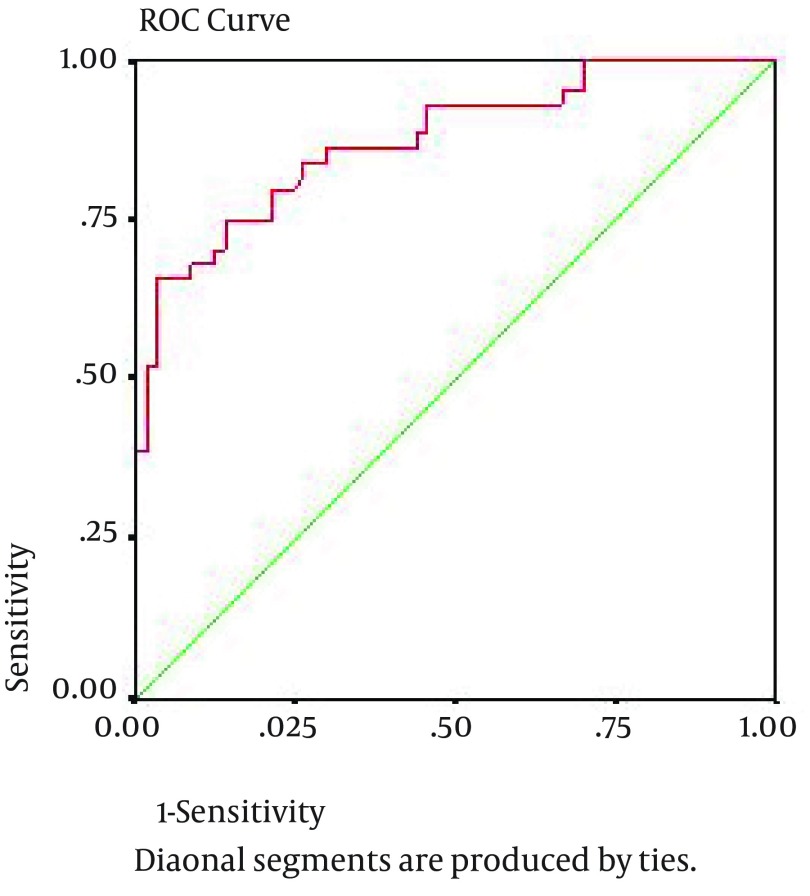
Sensitivity and Specificity of Vascular Endothelial Growth Factor Test in Receiver Operating Characteristic (ROC) Curve

Using ROC curve and area under curve (AUC) (Figure 1), we determined the sensitivity and specificity of VEGF as a diagnostic marker of prostate cancer. In the best cutoff point of VEGF = 188.2, sensitivity and specificity were 86.4% and 70.2%, respectively, and AUC was determined as 0.876. Odds ratio for such diagnosis was calculated as 14.9 with the 95%confidence interval of 3.89-57.1. moreover, calculating the sensitivity and specificity of the variable taken from the combination of VEGF and PSA as a diagnostic marker of prostate cancer (Figure 1), we determined a sensitivity of 91% and a specificity of 78% at the cutoff point of 102 and AUC was determined as 0.876. Odds ratio for such diagnosis was calculated as 35 with a 95% confidence interval of 6.3-194.2.

**Table 1.** Study Groups, Mean Ages, Vascular Endothelial Growth Factor, Prostate-Specific Antigen, and Prostate Volume Values ^a, b^

	Control	BPH	Cancer	P value
**Age, y**	67.2 ± 7.2	68.7 ± 8.3	66.5 ± 9.1	0.38
40-59	7 (12.3)	6 (10.5)	9 (20.5)	
60-69	26 (45.6)	25 (43.9)	24 (54.5)	
≥ 70	24 (42.1)	26 (45.6)	11 (25)	
**VEGF, pg/mL**	124.9 ± 50.8	147.5 ± 80	356.3 ± 181.1	< 0.001
**PSA, ng/mL**	1.94 ± 1.94	3.86 ± 2.3	38.7 ± 17.5	< 0.001
**Prostate volume**	37.4 ± 15.5	71.7 ± 25.5		< 0.001

^a^ Abbreviations: BPH, benign prostate hyperplasia; PSA, prostate-specific antigen; VEGF, vascular endothelial growth factor.

^b^Data are presented as mean ± SD or No. (%).

**Table 2.** Correlation Coefficient of Vascular Endothelial Growth Factor With Gleason Score, Prostate-Specific Antigen, and Prostate Volume ^a^

	Gleason Score	PSA	Prostate Volume
**Control**	-	r = - 0.01, P = 0.962	r = - 0.176, P = 0.381
**BPH**	-	r = 0.07, P = 0.61	r = - 0.059, P = 0.665
**Prostate cancer**	r = - 0.089, P = 0.566	r = 0.425, P = 0.004	-

^a^Abbreviations: BPH, benign prostate hyperplasia; PSA, prostate-specific antigen

## 5. Discussion

Morphology-based approaches, especially Gleason scoring, combined to clinical parameters of PSA and T stage have provided clinicians some important prognostic information about prostate cancer. Recent successes have served to cultivate the growing interest in discovering more molecular-based prognostic factors (2). Such biomarker should be quickly quantifiable in accessible biological fluids without any overlapping to untreated and healthy people; moreover, they should be consistent, cost-effective, readily interpretable by clinicians, prostate-specific, able to differentiate cancerous prostate and its stages from prostatic hyperplasia, evaluate the survival of the patients as well as response to treatment (18). VEGF, which induces vascular permeability and stimulates endothelial cell growth, is now recognized as a key factor required for growth of the tumors (6) and might be a prognostic factor in several tumors such as prostate cancer (19). It seems that VEGF has some of such above-mentioned advantages. According to our study, it may differentiate cancerous prostate from benign hyperplasia and according to Duque et al. study (7), it can discriminate metastatic disease from its localized form. In this regards, VEGF may resolve the drawback of PSA, which is tissue-specific rather than prostate cancer-specific and its serum concentration might be affected by several benign conditions (1, 18). Such false-positive results of PSA may unsubstantially necessitate further diagnostic evaluation, impose extra expenses, and lead to the employing more invasive procedures. It has been revealed that for every patient who benefits from PSA diagnosis-initiated treatment, 47 patients undergo unnecessary biopsy and other treatments because of false-positive PSA test results. Conversely, efforts to prevent such over diagnosis may lead to some delays in the treatment of aggressive and potentially life-threatening cancers (18). Some drawbacks of PSA are also related to its false-negative results. It has been demonstrated that prostate cancer may be detected in about 15% of men with normal or very low levels of total PSA, thereby making it difficult to reliably rule out the possibility of cancer at any PSA level (20). Considering that there are not reliable biomarkers as diagnostic as well as prognostic factors for prostate cancer (21), and in line with some other studies on tissue (10, 22) and serum (3), our study demonstrated that VEGF might be a potential biomarker for prostate cancer. According to some previous studies, however, there is not any consensus on the VEGF level expression as well as its prognostic and predictive value in prostate cancer and results are so controversial (22, 23). Such conflicting results could be due to different factors such as using different sample sizes, different stages of the cancer, and sensitivity of the used assays (22). Therefore, considering VEGF expression alone has a limited value in prostate cancer and according to our results, it seems that considering a combination of PSA and a proper cut-off point for VEGF might result in overcoming such drawbacks.

In line with Soulitzis et al. (15), we showed that serum VEGF might not be increased in prostatic hyperplasia. In addition, there were no statistical differences in serum VEGF level between BPH patients and healthy people in our study; a concept which was confirmed by some other studies (7) and is important to discriminate benign patterns from malignant ones. We suggest for the first time that VEGF combined with PSA, may be used as a powerful screening test to evaluate the risk of prostate cancer. We propose complementary researches using larger sample size to certificate the diagnostic validity of serum VEGF at different stages.

Our study had some advantages. First, relatively proper sample size and associated standard deviations yielded a proper power to detect differences in subgroup analyses. Second, all of our patients were newly diagnosed and there were no essential factors affecting the comparability of the groups including using different kinds of medical or surgical treatments. Such uniformity of the patients especially in the cancerous arm was seen less in other studies. In a case-control study, Vancleave et al. (24) showed that some alleles of VEGF gene have significant correlation with prostate cancer risk (P = 0.04). They supported the fact that genetics can affect VEGF expression. This means that VEGF may have different prognostic values in prostate cancer at different populations. Therefore, we should investigate the VEGF gene polymorphism in our population in the future studies.
